# Development of an Eye Irritation Test Method Using an In-House Fabrication of a Reconstructed Human Cornea-like Epithelium Model for Eye Hazard Identification

**DOI:** 10.3390/bioengineering11040302

**Published:** 2024-03-22

**Authors:** Naoki Yamamoto, Noriko Hiramatsu, Yoshinao Kato, Atsushi Sato, Hajime Kojima

**Affiliations:** 1 Support Office for Bioresource Research, Center for Translational Research, Translational Research Headquarters, Fujita Health University, Toyoake 470-1192, Aichi, Japan; norikoh@fujita-hu.ac.jp; 2 International Center for Cell and Gene Therapy, Research Promotion Headquarters, Fujita Health University, Toyoake 470-1192, Aichi, Japan; 3 Nippon Menard Cosmetic Co., Ltd., Nagoya 451-0071, Aichi, Japan; 4 Japanese Center for the Validation of Alternative Methods (JaCVAM), National Institute of Health Sciences (NIHS), Kawasaki 210-9501, Kanagawa, Japan; h-kojima@nihs.go.jp

**Keywords:** human corneal epithelial cell line (iHCE-NY1), Organization for Economic Co-operation and Development (OECD), reconstructed human cornea-like epithelium model

## Abstract

In a previous study, a novel human corneal-like epithelium model utilizing an immortalized human corneal epithelial cell line (iHCE-NY1) was developed as an alternative to animal models to identify chemicals not classified under the United Nations Globally Harmonized System of Classification and Labeling of Chemicals (GHS) and was evaluated following the criteria of Test Guideline 492 of the Organization for Economic Co-operation and Development (OECD). In the present study, our aim was to establish an eye irritation test protocol using the iHCE-NY1 model to classify liquid chemicals under the GHS ocular hazard categories: no effect, no classification (No Cat.), Category 2 (Cat. 2) reversible effects, and Category 1 (Cat. 1) irreversible eye damage. The protocol involved exposing the iHCE-NY1 model to 31 liquid test chemicals for 5 min, followed by observation at post-incubation periods (PIPs) to assess recovery. Classification was based on cell viability, and histopathological findings on PIP days 7, 14, and 21. The outcomes were compared with an established database of classifications. All Cat. 1 liquid chemicals, 62.5% of No Cat., and 63.2% of Cat. 2 were correctly categorized. This study demonstrates that the iHCE-NY1 model can not only distinguish No Cat. test liquid chemicals but also differentiate between Cat. 2 and Cat. 1 liquid chemicals.

## 1. Introduction

Historically, multiple agencies and regulatory bodies have used their own criteria to define which chemicals are hazardous to humans and to what extent. To bring these together into a unified global standard, the Globally Harmonized System of Classification and Labeling of Chemicals (GHS) established unified criteria. For example, pertaining to eye health, chemicals that cause irreversible damage to the eye are categorized as 1, hereafter referred to as Cat. 1, and those that cause reversible effects as Cat. 2, which may be further delineated into 2A, effects that fully reverse within 72 h, and 2B, those that fully reverse within 7 days. Substances that fall into neither Cat. 1 nor Cat. 2. do not require a GHS classification and are termed No Cat. [1].

Since the GHS does not dictate the test methods to be used to assign chemicals to categories, over the years, many have been proposed and tested including in vivo animal studies and in vitro eye models along with various protocols and assays to determine the effects of numerous chemicals. To promote the reliability and effectiveness of tests to assign substances into the GHS categories, the Organization for Economic Co-operation and Development (OECD) has prescribed test guidelines (TG) and reference chemicals that must be used. Developed in 1944, the Draize eye test, which is in accordance with OECD TG 405, has become the gold standard. It is an in vivo test using albino rabbits, which are typically euthanized after testing substances in their eyes. However, due to growing ethical concerns in society, several in vitro human eye models have been developed and vie to replace the classic in vivo test. While none have yet entirely replaced the in vivo test, some serve as initial steps to classify chemicals as No Cat., Cat. 1, or Cat. 2. Some methods can identify chemicals falling under Cat. 1 [2], while others identify those under No Cat. [3,4]. However, for some chemicals in Cat. 2, additional information is required to establish a definitive classification using these test methods.

Subsequently, the OECD has adopted test method TG492, which uses a reconstructed human cornea-like epithelium (RhCE) model [5], to serve as an in vitro model that can identify chemicals as No Cat. but not Cat. 2. However, another study presented an optimized SkinEthic™ human corneal epithelium (HCE) eye irritation test (EIT) method, applying the time-to-toxicity approach to test liquids and solids to distinguish between No Cat., Cat. 1, and Cat. 2. Based on existing SkinEthic™ HCE data and published data on a dilution approach with an RhCE model, different exposure times (5, 16, and 120 min) were selected, resulting in dose–response profiles instead of a single endpoint for each chemical [6,7]. The result was discussed in the OECD and the test method was approved as TG492B in 2022 [8]. Alternative testing strategies have been described in TG492B that combine the strengths of individual in vitro test methods to address the required ranges of irritant potential. Therefore, TG492B might be able to fully replace the Draize eye test method.

In a previous study by Kato et al. [9], a novel method was developed using an in-house fabrication of RhCE from an immortalized human corneal epithelial cell line (iHCE-NY1) [10,11] as an alternative to the SkinEthic™ human corneal epithelium eye irritation test method. Histopathological analysis of the iHCE-NY1 model, generated through this method, confirmed it represents a complete corneal epithelium comprising three distinct layers: the squamous epithelial layer, pterygium layer, and basal lamina. Furthermore, it validated that the eye irritation tests using this model meet the criteria of sensitivity, specificity, and accuracy outlined in the performance standard (PS) [12] of OECD Test Guideline 492 (TG492) [5] using the panel of 30 reference chemicals prescribed by the OECD.

The present study aimed to develop a test protocol capable of evaluating weak to moderate eye irritants in liquid form using iHCE-NY1. This involved exposing the iHCE-NY1 model to 31 test chemicals for 5 min, then making observations for effects at post-incubation periods (PIPs) of 7, 14, and 21 days. Classification was assigned based on observed cell viability using the WST-8 assay where the mitochondria of viable cells affects a chemical that, after processing, allows for colorimetric detection wherein the measure of color is directly proportional to the number of viable cells.

Additionally, histopathological analysis was performed to evaluate cell proliferation. The resulting classifications were compared to the predicted classifications in a reference database. The classifications represent predictions of how the substances tested in vitro would affect real eyes.

## 2. Materials and Methods

### 2.1. Test Substances

This test method was evaluated using 31 test chemicals including 15 liquid chemicals listed as reference chemicals in Table 1 based on TG492 PS [12]. The selection of test chemicals aimed to represent four GHS categories: four Cat. 1, twelve Cat. 2A, seven Cat. 2B, and eight No Cat. (totaling 31). These chemicals were sourced from AK Scientific (Union City, CA, USA), Fujifilm Wako Chemicals (Tokyo, Japan), Sigma-Aldrich (St. Louis, MO, USA), and Tokyo Chemical Industry (Tokyo, Japan) (see Table 1). Negative and positive controls utilized 3-methoxy-1,2-propanediol (Sigma-Aldrich) and benzalkonium chloride (Fujifilm Wako Chemicals), respectively.

### 2.2. Development of iHCE-NY1 Model

#### 2.2.1. Cells and Culture Conditions

The cell line employed in this study was the immortalized human corneal epithelial cell line iHCE-NY1 as described by Yamamoto et al. [10,11]. The cell line, derived from human corneal tissue and transfected with SV40 large T antigen [13,14], is available from RIKEN Cell Bank, Ibaraki, Japan. Cultures were maintained in Dulbecco’s modified Eagle’s medium/F12 medium (Sigma-Aldrich), supplemented with 5% fetal bovine serum (Sigma-Aldrich), 100 U/mL penicillin (Sigma-Aldrich), 100 μg/mL streptomycin (Sigma-Aldrich), 5 μg/mL insulin–transferrin–selenium (Invitrogen; Thermo Fisher Scientific, Inc., Waltham, MA, USA), 0.5% dimethyl sulfoxide (Sigma-Aldrich), 10 ng/mL epidermal growth factor (Sigma-Aldrich), and 0.5 μg/mL hydrocortisone (Sigma-Aldrich) at 37 °C and 5% CO_2_ in a humidified incubator. Cells were harvested using TrypLE™ Select (Invitrogen; Thermo Fisher Scientific, Inc.); when they reached 70–80% confluence, they were sub-cultured at a density of 0.5–1 × 10^4^ cells/mL. The constructed RhCE (iHCE-NY1 model) was derived from passages 30 to 65 of the cell line.

#### 2.2.2. Construction

Initially, 700 μL of the cell culture medium was dispensed into each well of a companion plate (Falcon 353504; Becton, Dickinson and Company, Franklin Lakes, NJ, USA), followed by placing a cell culture insert (Falcon 353495) in each well. The iHCE-NY1 cells were then harvested from the culture and seeded onto each cell culture insert with 200 μL of cell suspension (3 × 10^5^ cells/mL). Subsequently, after a submerged culture period of 3 days, the growth medium in the companion plate wells was replaced with fresh medium (350 μL per well), while the culture medium within the cell culture insert was carefully removed to avoid damaging the cells. The cell culture insert was attached to the wells of the companion plate with the upper surface exposed to air, initiating an air–liquid interface culture (air lift culture) that was then incubated for 10 days. Throughout the air lift culture period, the growth medium in the plate wells (350 μL per well) was replaced every 2 or 3 days, and the histopathology of the base model was examined before treatment.

#### 2.2.3. Viability

Cell viability was assessed using the WST-8 assay, which measures the reduction in WST-8 by viable cells in the iHCE-NY1 model. Tissues were cultured on 24-well plates containing 300 μL of freshly prepared WST-8 solution (1:10 dilution of Cell Counting Kit-8 [Dojindo, Kumamoto, Japan] with cell culture medium). Following 3 h incubation under standard cultivation conditions, 100 μL of the culture supernatant was transferred to a 96-well microtiter plate. The absorbance of the culture supernatant was then measured at wavelengths of 450 nm and 650 nm as a reference absorbance using a microplate reader (VersaMax; Molecular Devices, LCC, San Jose, CA, USA), with the WST-8 solution serving as a blank. Suitable ranges for the iHCE-NY1 model were determined for each batch based on a non-treated control.

#### 2.2.4. Morphology

The iHCE-NY1 model was fixed in SUPER FIX [10] and a paraffin block was prepared as usual and subjected to histopathological examination. Vertical sections of 5 µm were cut, stained with hematoxylin and eosin, and examined under a light microscope. For BrdU labeling to be incorporated into the S phase of the cell cycle, a 10 µM BrdU labeling medium was prepared by diluting a 10 mM BrdU stock solution (ab142567, Abcam plc., Cambridge, UK) with medium. The existing culture medium was removed from the iHCE-NY1 models and replaced with the 10 µM labeling medium. The iHCE-NY1 models were then incubated in the BrdU labeling medium for 12 h at 37 °C in a CO_2_ incubator. Subsequently, the BrdU labeling medium was removed from the cells, which were then washed twice in 10 mL of Dulbecco’s phosphate-buffered saline (DPBS) (Sigma-Aldrich). Fixed and paraffin sections were prepared, and BrdU was detected using a sheep polyclonal anti-BrdU antibody (1:100, Capralogics, Hardwick, MA, USA) and an anti-sheep IgG antibody labeled with Alexa Fluor 594 (1:1000, Thermo Fisher Scientific Inc., Waltham, MA, USA). DAPI (Vectashield H-1200; Vector Laboratories, Burlingame, CA, USA) was used for nuclear staining. A fluorescence microscope (Power BX-51; Olympus, Tokyo, Japan) was used for observation.

### 2.3. Test Protocol and Cell Viability in Tested iHCE-NY1 Models

#### 2.3.1. Test Protocol

Treatment with the test chemicals and control substances involved the use of at least two tissue replicates for each test chemical and each control substance in each run. For the liquid test chemicals, two models were treated with 25 μL of the test chemical. Additionally, two models were treated with 25 μL of 3-methoxy-1,2-propanediol as negative controls, two tissues were treated with 25 μL of a 10% solution of benzalkonium chloride as positive controls, and two models were left untreated as untreated controls. The tissues were then incubated for 5 min, which was considered to be the minimum exposure time required to eliminate errors during the measuring operation. After the 5-min exposure period, each model was rinsed thrice or more with 10 mL of DPBS using an electronic pipette to remove any residual test chemical from the model surface. The tissues were blotted and transferred to new wells in 24-well plates containing 700 μL of fresh cell culture medium. The model was then incubated under standard cultivation conditions for 24 h. Following the incubation period, the tissues were blotted and transferred to new wells in 24-well plates containing 300 μL of freshly prepared WST-8 solution and incubated for 3 h under standard cultivation conditions. Subsequently, 100 μL of the culture supernatant or WST-8 solution was transferred to a 96-well microtiter plate. The absorbance value of the culture supernatant was measured at 450 nm and 650 nm as a reference absorbance, using the WST-8 solution as a blank. Cell viability was calculated as a percentage relative to the viability of the untreated control. The mean of the individual three values from identically treated tissues was used to determine a prediction according to the prediction model [9].

A test chemical was categorized as No Cat., not requiring classification and labeling according to the GHS, if the mean percentage cell viability after exposure and PIP exceeded the established percentage cell viability cut-off value of 70% (EC70), as determined by the prediction model.

#### 2.3.2. Cell Viability in Tested iHCE-NY1 Models

**Figure 1:**
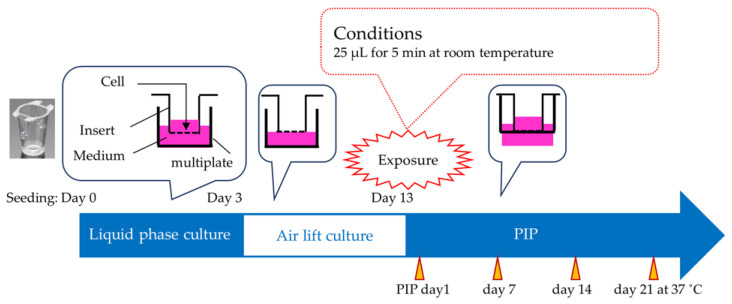
Fabricating and testing with an RhCE (iHCE-NY1) model. PIP: post-incubation period. RhCE: reconstructed human cornea-like epithelium.

**Figure 2:**
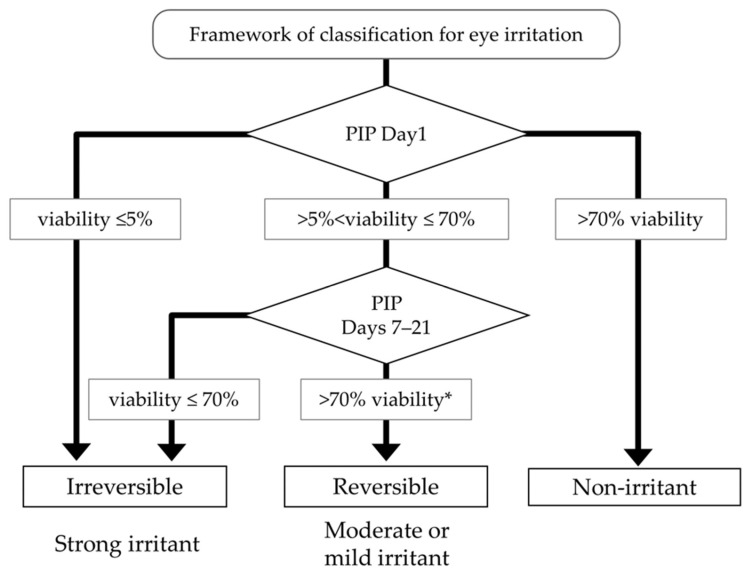
Classification framework for eye irritation. PIP: post-incubation period. * If time-dependent increase in viability is observed and viability is greater than 70%.

The exposure duration for the liquid test chemicals was 5 min, followed by 1 day PIP, while that for the solid test chemicals was 6 h followed by 18 h PIP. In this study, undiluted test chemicals were directly applied to the models. This protocol for recovery analysis was incorporated utilizing Cat. 2 test chemicals. Test chemicals from different eye irritation categories were applied to the model, and the recoverability of EC70 at PIPs 7, 14, and 21 days was assessed based on cell viability and histopathology findings (Figure 1). Cell viability of the tissues was evaluated immediately after the PIP. Tissues with viability between >5% and ≤70% on the first day of PIP were further monitored for cell viability at PIP days 7 and 14, as shown in Figure 2. Additionally, histopathology specimens of the tissues were prepared concurrently with the cell viability measurements to confirm any histopathological changes.

### 2.4. Predictive Capacity and Recovery in iHCE-NY1 Models

Reversible and irreversible effects were assessed for the 31 test chemicals. Predictive capacity was determined based on PIP days 7, 14, and 21 using data derived from the Draize eye test. Performance statistics were reported for weighted predictions compared to reference data obtained from the Draize eye test as described [15,16].

## 3. Results

### 3.1. Predictive Capacity with a PIP of 1 Day

The cell viabilities of the positive and negative controls were correctly categorized in all experiments (Supplementary Figure S1). Among the 23 Cat. 1 and Cat. 2 (2A and 2B) test chemicals as well as the eight No Cat. test chemicals, 20 exhibited viabilities ≤ 70%, and were classified as irritants (Table 2). The remaining test chemicals exhibited viabilities > 70% and were classified as non-irritants (Table 1).

The sensitivity and specificity of the predictions were calculated as 87.0% (20/23) and 62.5% (5/8), respectively, with an overall accuracy of 80.6% (25/31) (Table 2). While the overall accuracy met the criteria specified in the TG492 PS, the sensitivity did not (the criteria being sensitivity 90%, specificity 60%, and accuracy 75%). The sensitivity was affected by the increased classification of 16 Cat. 2 test chemicals and one Cat. 1 (Table 2), posing challenges in predicting Cat. 2 test chemicals.

For the solid test chemicals, the predictive capacity was evaluated with 16 test chemicals including 13 based on TG492 PS (Supplementary Table S1). While the sensitivity of the protocol met 100% (9/9), the specificity was 57.1% (4/7), failing to meet the TG492 PS criteria (Supplementary Table S2b). Consequently, the physical state of the test chemical emerged as a crucial factor influencing predictive capacity, leading to the decision to focus solely on evaluations with liquid chemicals. Further optimization is necessary to enhance the predictive capacity for solid test chemicals. The results for liquid and solid PIP1 for 47 substances are presented in Supplementary Table S2a.

### 3.2. Effect of PIPs 7 to 21 Days on Cell Recovery

#### 3.2.1. Recovery of Cell Viability

**Figure 3:**
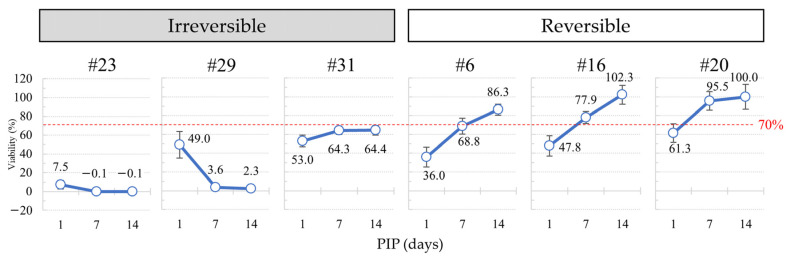
Some examples on the recovery of cell viability with the WST-8 assay. Cell viability was measured post-incubation period (PIP) 1, 7, and 14 days, and cell viability was converted to 100% of that of the negative control. Cell viability greater than 50% of that of the negative control was used as the recovery criterion (purple dotted line). List of test chemicals: #23: n-butanal, #29: ethyl thioglycolate, #31: polyethylene glycol (PEG-40) hydrogenated castor oil, #6: γ-butyrolactone, #16: 2-methyl-1-pentanol, #20: ethyl-2-methylacetoacetate.

Seven test chemicals, three Cat. 1, three Cat. 2, and one No Cat., were classified as strong irritants/irreversible as the cell viability after a PIP of 1 day was ≤5% (see Figure 2). Conversely, eight liquid chemicals including five No Cat. and three Cat. 2 were classified as non-irritants, showing viability ≥ 70% after 5 min exposure and 24 h PIP (Table 1). The remaining 16 test chemicals, with viabilities ranging between >5% and ≤70%, underwent further culture up to a maximum of PIP 21 days. Subsequently, all showed recovery with a PIP of 14 days. Among these, 12 test chemicals were classified as moderate or mild irritants. Notably, all 12 belonged to Cat. 2 (Table 1 and Supplementary Figure S2). For example, the recovery patterns of #6: γ-butyrolactone (Cat. 2A), #16: 2-methyl-1-pentanol (Cat. 2B), and #20: ethyl-2-methylacetoacetate were observed, while the non-recovery patterns of #23: n-butanal, #29: ethyl thioglycolate, and #31: polyethylene glycol (PEG-40) hydrogenated castor oil were evident (Figure 3). The remaining four chemicals, one Cat. 1, one Cat. 2, and two No Cat., were deemed irreversible as their viability did not exceed 50% with a PIP of 14 days. Consequently, these chemicals were classified as strong irritants.

To summarize the recovery of cell viability, all four Cat. 1, four Cat. 2, and three No Cat. test chemicals were classified as strong irritants, as indicated in Table 1 and Supplementary Figure S2. Additionally, 12 Cat. 2 test chemicals were classified as moderate or mild irritants. Conversely, three Cat. 2 and five No Cat. test chemicals were classified as non-irritants, as shown in Table 1.

#### 3.2.2. Histopathological Findings

**Figure 4:**
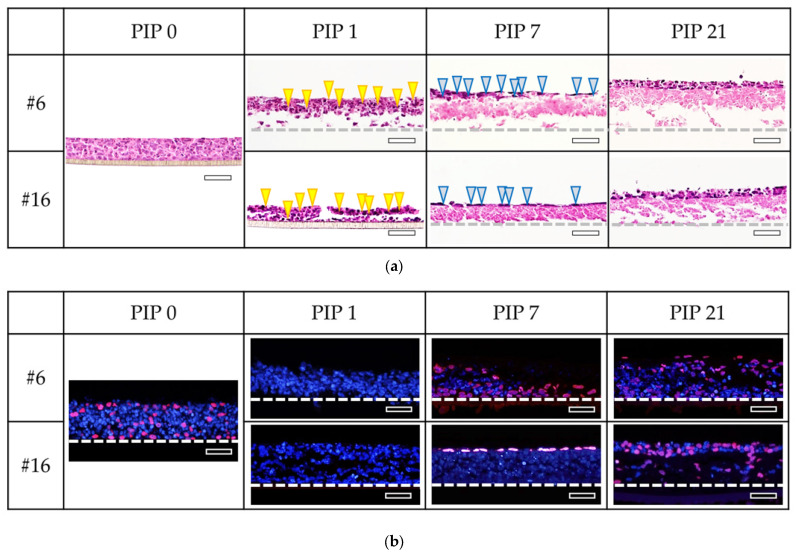
(**a**) Monitoring the recovery of the iHCE-NY1 model using histological cross-sections. HE-stained tissue specimens of the iHCE-NY1 model’s incubation for 1- (post-incubation period (PIP) 1), 7-(PIP 7), 14-(PIP 14), and 21-days (PIP 21) post exposure to liquid chemicals are shown. At PIP 1, cells with enriched nuclei (yellow arrowheads) were observed, while viable cells (blue arrowheads) were observed from PIP 7. As the culture days increased to PIP 14 and PIP 21, more viable cells were observed. The gray dotted line indicates the position of the bottom of the iHCE-NY1 model. #6: γ-butyrolactone, #16: 2-methyl-1-pentanol, all bar indicates 50 µm. (**b**) Detection of respirating cells in the iHCE-NY1 model. Cells in S phase of the cell cycle that took up BrdU had cell nuclei revealed with red fluorescence. At post-incubation period (PIP) 1-day, few cells were in the S phase. From PIP 7-day onward, BrdU-positive S-phase cells were detected in all samples. #6: γ-butyrolactone, #16: 2-methyl-1-pentanol, all bar indicates 50 µm.

Histopathological examination of two test chemicals (#6, γ-butyrolactone and #16, 2-methyl-1-pentanol) obtained during the recovery response revealed a significant increase in the number of epithelial layers at 21 days, as depicted in Figure 4a. BrdU, a marker of cell proliferation, was utilized to assess cell activity, as illustrated in Figure 4b. The immunostaining of BrdU indicated a significant increase in the number of epithelial cells at 21 days. Furthermore, to confirm the 3D nature of the cornea model, immunostaining results for keratin 3, a marker specific to corneal cells, and involucrin, which is observed in intercellular junctions in the 3D model, are presented (Supplementary Figure S3).

#### 3.2.3. Table (3 × 3) by PIPs 7 to 14 Days

**Figure 5:**
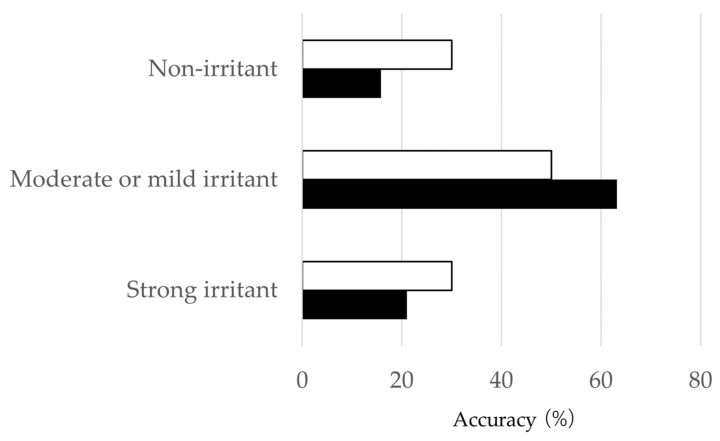
Accuracy for the assessment of UN GHS Category 2 by EIT recovery using the iHCE-NY1 model test method. Open column: OECD performance criteria, black column: predictivity of EIT recovery using the iHCE-NY1 model test method for liquids. Balanced accuracy: 67.7%.

The predictive capacities of four Cat. 1 test chemicals, twelve Cat. 2 test chemicals, and five No Cat. test chemicals were 100% (4/4), 63.2% (12/19), and 62.5% (5/8), respectively (Supplementary Table S3a). These data met the criteria for the Cat. 1 and Cat. 2 test chemicals but not for No Cat., according to the OECD performance standards (Cat. 1, ≥75%; Cat. 2, ≥50%; No Cat., ≥70%) [8]. The balanced accuracy limited to liquids was 67.7% (21/31) for the iHCE-NY1 model, as shown in Figure 5. The balanced accuracy decreased to 63.8% (30/47) for both liquids and solids, and to 56.3% (9/16) for solids alone compared to the overall accuracy (Supplementary Table S3b,c).

## 4. Discussion

In this study, we assessed the ability of a test protocol using the in vitro iHCE-NY1 model as a proxy for established in vivo methods to predict how hazardous liquid substances are for human eyes. It is predictive because the classifications were determined in an in vitro model representing a real eye. The obtained classifications for particular substances were compared with those in an established database formulated from the results of numerous independent in vitro studies. The predictivity of the protocol was assessed, which is the ability to distinguish between different hazard classifications compared to classifications in the database. Performance values were determined, which specify the minimum percentage of correct predictions required for each category in order for the protocol to be considered effective. The performance values should be at least 75% for Cat. 1, 50% for Cat. 2, and 70% for No Cat. substances [8].

The WST-8 assay conducted to quantify the recovery of cell viability in the iHCE-NY1 model is important because cell viability is a key parameter in understanding the mechanism of eye irritation [17,18]. In the TG492 guidelines [5], the tetrazolium dye (MTT, WST-8, or WST-1) assay is primarily employed to assess cell or tissue viability. However, the MTT assay produces insoluble formazan, requiring the use of a solvent in its measurements, which necessitates the destruction of tissue [19], whereas WST-8 and WST-1 enable the viable cells of the tissue construct to reduce the dye into a water-soluble form, facilitating non-destructive measurement. The non-destructive WST-8 assay was adopted to determine tissue viability as it allows for continued culture of the tissues post-assay. It is important to clarify that while commercial kits of the WST-8 cell proliferation assay are available, the WST assay primarily measures mitochondrial activity and cellular respiration, rather than actual cell proliferation (i.e., mitosis). Nevertheless, the WST assay, similar to MTT, offers a means to monitor tissue viability continuously throughout the recovery period (PIP 1 to 14 days).

Additionally, for chemicals that induced reversible tissue damage, we assessed tissue recovery using histopathology. Upon extending the PIP from 7 to 21 days, the histopathological findings confirmed cell proliferation. The proliferated cells in the basal layer indicated recovery (Figure 4b). Comparison of histopathological images of #6, γ-butyrolactone and #16, 2-methyl-1-pentanol at PIP day 7 revealed that γ-butyrolactone exhibited a lack of cells in the basal layer. Since iHCE-NY1 originated from marginal stem cells, it possessed high differentiation potential, meaning that the cells in the model may have the capacity to develop into different types of cells, which may have contributed to the model’s ability to recover from damage.

The framework for the classification of eye irritation presented in this paper is shown in Figure 2, and the recovery of tissue viability on given PIPs 7 to 14 days is detailed in Table 1 and Supplementary Table S3a. The balanced accuracy for predictions made using this test was 67.7% (21/31), as depicted in Figure 5. Among the 19 Cat. 2 chemicals, four were overestimated, false positive (21.0%, 4/19) as strong irritants, and three were underestimated, false negative (15.8%, 3/19) as non-irritants. Additionally, among the eight No Cat. chemicals, three were overestimated, false positives (37.5%, 3/8) as strong irritants. We considered the framework <would meet the criteria/was similar to the level> of TG492B because its predictive capacity of Cat. 2 chemicals was ≥50%.

However, the predictivity did not meet the performance requirements of TG492B [8], and false positive results were observed for chemicals categorized as Cat. 2 and No Cat. Specifically, three No Cat. chemicals (#26, 3-phenoxybenzyl alcohol, #29, ethyl thioglycolate, and #31, polyethylene glycol (PEG-40) hydrogenated castor oil; as shown in Table 1) did not meet the performance criteria. Among these, #29 and #31 were classified as inducing irreversible viability, consistent with the results from other corneal tissue test methods such as EpiOcular™ EIT, SkinEthic™ HCE EIT, LabCyte CORNEA-MODEL24 EIT, and MCTT HCE™ EIT [12,20,21]. These false positives were attributed to the protocol of PIP 1-day. The prediction model has been developed to reduce the false negatives in the OECD performance criteria because this assay is available for regulatory use. Therefore, we think that some false positives (overestimating results) are within the acceptable range.

Furthermore, some non-irritant test chemicals did not meet the OECD performance criteria, likely because the number of No Cat. candidates was only 15 (Supplementary Table S3a), and these false negatives were similarly associated with the protocol of PIP 1-day. Among the Cat. 2 test chemicals, three were underestimated as false negatives in this assay: #10, methyl cyanoacetate, #19, 3-chloropropionitrile, and #22, isopropyl acetoacetate, as shown in Table 1. The eye irritation potential of #10 and #22 were classified as in vitro non-irritant because conjunctival and iris damage was observed at PIP 72 h [15,22]. Additionally, #19 was categorized as Cat. 2B due to corneal damage; however, in another study with this chemical, one of three rabbits exhibited no corneal damage while the remaining two initially showed damage. However, by PIP 72 h, the animals had recovered and exhibited only extremely weak eye irritation [22]. With an extended PIP, no additional false negative test chemicals were observed. Ultimately, there was no difference in recovery between the Cat. 2A and 2B test chemicals. However, some substances could be identified as Cat. 2, but could not be further classified into 2A or 2B in this study.

The present study has some limitations. The performance of the protocol for solids was insufficient. As such, to predict the GHS categories of solid chemicals, we intend to reexamine the method protocols or devise a different set for solids to achieve better predictivity in the future (Supplementary Table S3b). Although we propose the protocol used here as an alternative to existing in vitro test methods, we did not directly compare it with existing methods as we are still in the exploratory phase of its development. This model is a qualitative rather than quantitative assay, and the sensitivity was similar compared to other commercially available models such as EpiOcular and SkinEthic RHE. The recoverability of this model by WST-8 could be verified by PIP 14. On the other hand, recoverability by cell proliferation using tissue specimens was confirmed by PIP 21, which was extended for another 7 days. However, the model broke down like other commercial models when the culture was extended beyond PIP 21, so we did not assess the long-term effects beyond PIP 21. Furthermore, we did not examine whether the optical properties of the tested chemicals could directly affect the reduction in WST-8 or indirectly affect the color outcomes. Finally, the number of chemicals tested was close to the minimum required.

## 5. Conclusions

In conclusion, testing liquid substances to determine whether they are hazardous for eyes using the iHCE-NY1 model shows promise as an alternative to using animals. This model can be easily and inexpensively fabricated in a laboratory as an alternative to purchasing an expensive model. The present study evaluated the predictive capacity of the iHCE-NY1 model, which has the potential to distinguish between No Cat., Cat. 2, and Cat. 1 test chemicals for liquids according to the GHS of Classification and Labeling of Chemicals as well as the test method outlined in OECD TG492B. Implementing this assay could simplify the screening for eye irritation worldwide without the need for animal testing.

## Figures and Tables

**Table 1:** List of test chemicals and results of the iHCE-NY1 model test method for liquids.

No.	CAS RN	Chemical Name	Supplier	OECD	In Vivo Category	In Vitro Prediction	Judgment: Recoverability	In Vitro Prediction with PIP
				Original TG492PS	(UN GHS)			
1	1760-24-3	(Ethylenediamine-propyl)-trimethoxysilane	Sigma-Aldrich	〇	1	Irritant	NT	Strong
2	2365-48-2	Methylthioglycolate	Sigma-Aldrich	〇	1	Irritant	Irreversible	Strong
3	818-61-1	Hydroxyethyl acrylate	FUJIFILM Wako	〇	1	Irritant	NT	Strong
4	17831-71-9	Tetraethylene glycol diacrylate	Sigma-Aldrich		1	Irritant	NT	Strong
5	18472-51-0	2,4,11,13-Tetraazatetradecane-diimidamide, N,N″-bis(4-chlorophenyl)-3,12-diimino-, di-D-gluconate (20%, aqueous)	Sigma-Aldrich	〇	2A	Irritant	NT	Strong
6	96-48-0	γ-Butyrolactone	Sigma-Aldrich	〇	2A	Irritant	Reversible	Moderate or mild
7	104-76-7	2-Ethyl-1-hexanol	FUJIFILM Wako		2A	Irritant	NT	Strong
8	67-64-1	Acetone	Sigma-Aldrich		2A	Irritant	Reversible	Moderate or mild
9	67-63-0	Isopropyl alcohol	FUJIFILM Wako		2A	Irritant	Reversible	Moderate or mild
10	105-34-0	Methyl cyanoacetate	Sigma-Aldrich		2A	Non-irritant	NT	Non-irritant
11	78-93-3	Methyl ethyl ketone (2-butanone)	TCI		2A	Irritant	Reversible	Moderate or mild
12	111-27-3	n-Hexanol	Sigma-Aldrich		2A	Irritant	Reversible	Moderate or mild
13	96-41-3	Cyclopentanol	Sigma-Aldrich		2A	Irritant	Reversible	Moderate or mild
14	1569-01-3	Propylene glycol propyl ether	Sigma-Aldrich		2A	Irritant	Reversible	Moderate or mild
15	9002-93-1	Triton X-100 (5%)	Sigma-Aldrich		2A	Irritant	Reversible	Moderate or mild
16	105-30-6	2-Methyl-1-pentanol	Sigma-Aldrich	〇	2B	Irritant	Reversible	Moderate or mild
17	134-62-3	Diethyl toluamide	Sigma-Aldrich	〇	2B	Irritant	Reversible	Moderate or mild
18	29911-27-1	1-(2-Propoxy-1-methylethoxy)-2-propanol	Sigma-Aldrich		2B	Irritant	Reversible	Moderate or mild
19	542-76-7	3-Chloropropionitrile	FUJIFILM Wako		2B	Non-irritant	NT	Non-irritant
20	609-14-3	Ethyl-2-methylacetoacetate	Sigma-Aldrich		2B	Irritant	Reversible	Moderate or mild
21	78-84-2	Isobutyraldehyde	Sigma-Aldrich		2B	Irritant	NT	Strong
22	542-08-5	Isopropyl acetoacetate	FUJIFILM Wako		2B	Non-irritant	NT	Non-irritant
23	123-72-8	n-Butanal	FUJIFILM Wako		2B	Irritant	Irreversible	Strong
24	342573-75-5	1-Ethyl-3-methylimidazolium ethylsulfate	Sigma-Aldrich	〇	NC	Non-irritant	NT	Non-irritant
25	2370-63-0	2-Ethoxyethyl methacrylate	Sigma-Aldrich	〇	NC	Non-irritant	NT	Non-irritant
26	13826-35-2	3-Phenoxybenzyl alcohol	Sigma-Aldrich	〇	NC	Irritant	NT	Strong
27	3446-89-7	4-(Methylthio)-benzaldehyde	Sigma-Aldrich	〇	NC	Non-irritant	NT	Non-irritant
28	629-19-6	Dipropyl disulfide	Sigma-Aldrich	〇	NC	Non-irritant	NT	Non-irritant
29	623-51-8	Ethyl thioglycolate	Sigma-Aldrich	〇	NC	Irritant	Irreversible	Strong
30	51-03-6	Piperonyl butoxide	Sigma-Aldrich	〇	NC	Non-irritant	NT	Non-irritant
31	61788-85-0	Polyethylene glycol (PEG-40) hydrogenated castor oil	FUJIFILM Wako	〇	NC	Irritant	Irreversible	Strong

FUJIFILM Wako: FUJIFILM Wako Pure Chemical Corporation, TCI: Tokyo Chemical Industry Co., Ltd., 〇: Reference chemicals listed in TG492 performance standard (PS), NT: Not tested.

**Table 2:** Outcomes of the eye irritation evaluation using the iHCE-NY1 model test method (PIP: 1 day).

Liquid Chemicals		In Vivo Category (UN GHS)		Number of Chemicals
		Category 1, 2A and 2B	No Category	
In vitro prediction	Irritant (viability ≤ 70%)	20	3	23
	Non-irritant (viability > 70%)	3	5	8
Number of chemicals		23	8	31

Sensitivity: 87.0%, specificity: 62.5%, accuracy: 80.6%. OECD criteria: sensitivity: 90.0%, specificity: 60%, accuracy: 75%. PIP: post-incubation period.

## Data Availability

The data are presented in this study.

## Supplementary Materials

The following supporting information can be downloaded at: https://www.mdpi.com/article/10.3390/bioengineering11040302/s1, Figure S1: Historical data of viability on day-1 post culture of cornea model treated with the negative control (3-methoxy-1,2-propanediol); Figure S2: Recovery of cell viability in the WST-8 assay for 16 liquid test chemicals that needed a post-incubation period (PIP) of 1–14 days to allow subcategorization of the eye irritation evaluation using the iHCE-NY1 model test method; Figure S3: Corneal epithelial cell marker keratin 3 and involucrin; Table S1: The list of test chemicals and results of the iHCE-NY1 model test method for solids; Table S2: (a) The outcome of the eye irritation evaluation using the iHCE-NY1 model test method (PIP: 1 day) for liquids and solids; (b) The outcome of the eye irritation evaluation using the iHCE-NY1 model test method (PIP: 1 day) for solids; Table S3: (a) Performance metrics for assessment of the eye irritation evaluation using the iHCE-NY1 model test method for liquids in accordance with OECD criteria and this assay for eye hazard identification; (b) The outcome of the eye irritation evaluation using the iHCE-NY1 model test method (PIP: by 14 days) for liquids and solids; (c) The outcome of the eye irritation evaluation using the iHCE-NY1 model test method (PIP: by 14 days) for solids.
