# Supplementary material for: Development of an Eye Irritation Test Method Using an In-House Fabrication of a Reconstructed Human Cornea-like Epithelium Model for Eye Hazard Identification
Source: Bioengineering (Basel). 2024 Mar 22;11(4):302. doi: 10.3390/bioengineering11040302 (PMC11047593; doi:10.3390/bioengineering11040302)
Supplement: Supplementary file 1 [file bioengineering-11-00302-s001.zip › bioengineering-2922265-supplementary.pdf]

bioengineering

MDPI

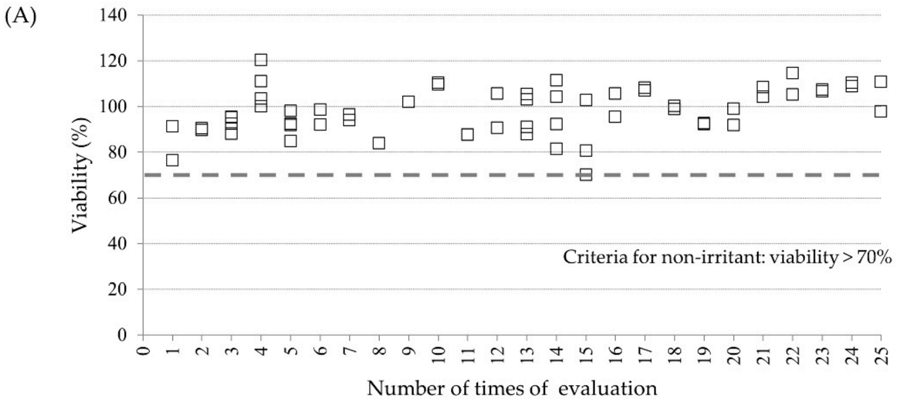

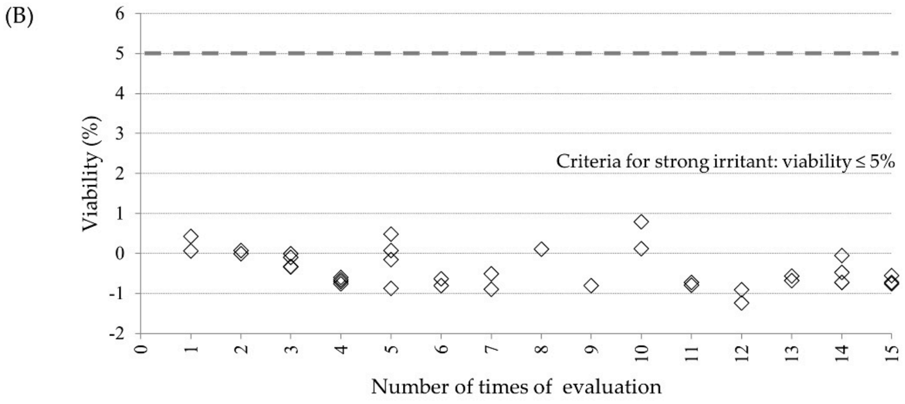

Figure S1. Historical data of viability on day-1 post culture of cornea model treated with the negative control (3-methoxy-1,2-propanediol). (A) Historical data of viability on day-1 post culture of cornea model treated with the negative control (3-methoxy-1,2-propanediol). (B) Historical data of viability on day-1 post culture of cornea model treated with the positive control (10% benzalkonium chloride solution).

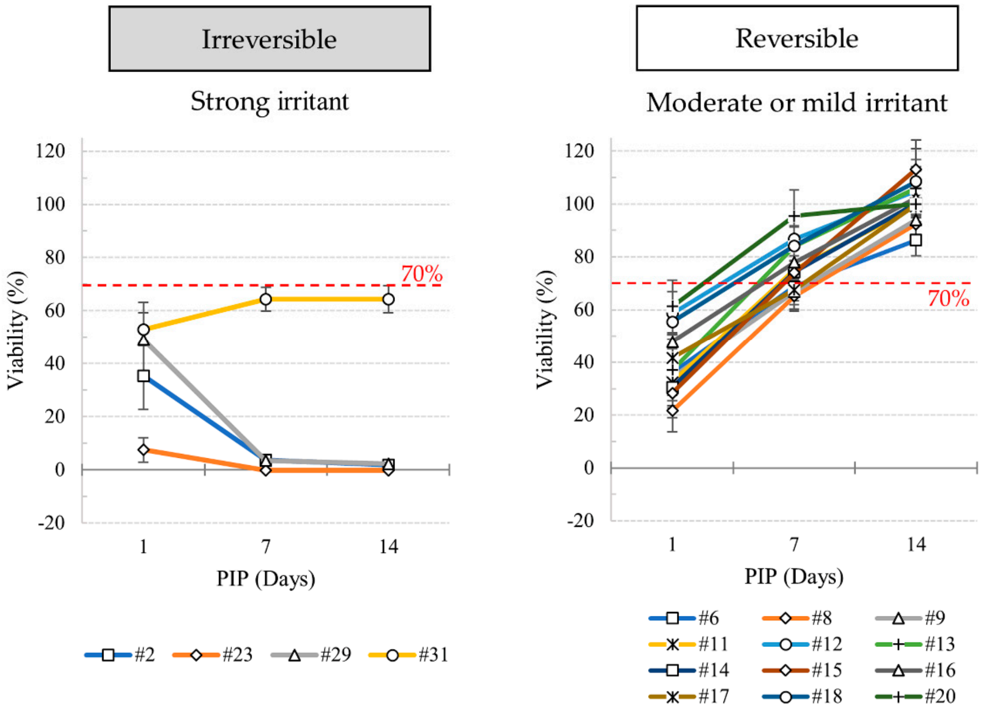

Figure S2. Recovery of cell viability at WST-8 assay for 16 liquid test chemicals which needed post-incubation period (PIP) of 1–14 days to allow subcategorization of the eye irritation evaluation using the iHCE-NY1 model test method.

List of chemicals:

#2: Methylthioglycolate, #23: n-Butanal, #29: Ethyl thioglycolate, #31: Polyethylene glycol (PEG-40) hydrogenated castor oil.

#6: γ-Butyrolactone, #8: Acetone, #9: Isopropyl alcohol, #11: Methyl ethyl ketone (2-butanone), #12: n-Hexanol, #13: Cyclopentanol, #14: Propylene glycol propyl ether, #15: Triton X-100 (5%), #16: 2-Methyl-1-pentanol, #17: Diethyl toluamide, #18: 1-(2-Propoxy-1-methylethoxy)-2-propanol, #20: Ethyl-2-methylacetoacetate.

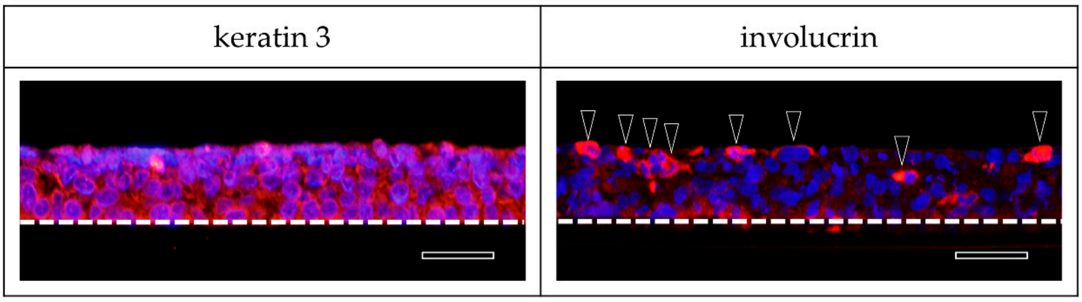

Figure S3. Corneal epithelial cell marker keratin 3 and involucrin. Corneal epithelial cell marker keratin 3 was detected. In addition, involucrin was detected in the relatively superficial layers. This is a three-dimensional model that mimics the corneal epithelium. Primary antibodies used were anti-keratin 3 antibody (CBL218, Merck KGaA, Germany) or anti-involucrin antibody (ab68, abcam). Alexa Fluor 594 antibody was used as a secondary antibody.

Table S1. The list of test chemicals and results of iHCE-NY1 model test method for solid.

No.	CAS No.	Chemical Name	Supplier	OECD Original TG492PS	*In vivo* category (UN GHS)	*In vitro* prediction	Judgment: Recoverability
**32**	**2634-33-5**	**1,2-Benzisothiazol-3(2H)-one**	**FUJIFILM Wako**	○	1	Irritant	NT
33	110-03-2	2,5-Dimethyl-2,5-hexanediol	Sigma-Aldrich	○	1	Irritant	NT
34	27344-41-8	Disodium 2,2'-([1,1'-biphenyl]-4,4'-diyldivinylene)bis-(benzenesulphonate)	FUJIFILM Wako	○	1	Irritant	NT
35	62-76-0	Sodium oxalate	Sigma-Aldrich	○	1	Irritant	NT
36	16867-03-1	2-Amino-3-hydroxy pyridine	Sigma-Aldrich		2A	Irritant	
37	83-56-7	1,5-Naphthalenediol	TCI		2A	Irritant	
38	532-32-1	Sodium benzoate	Sigma-Aldrich	○	2A	Irritant	
39	104-36-9	1,4-Dibutoxy benzene	TCI	○	2B	Irritant	Reversible
40	79-92-5	2,2-Dimethyl-3-methylenebicyclo [2.2.1] heptane	Sigma-Aldrich	○	2B	Irritant	
41	101-20-2	1-(4-Chlorophenyl)-3-(3,4-dichlorophenyl) urea	Sigma-Aldrich	○	NC	Non-irritant	NT
42	3179-89-3	2,2'-[[3-Methyl-4-[(4-nitrophenyl)azo]-phenyl]imino]bis-ethanol	AK Science		NC	Irritant	
43	103597-45-1	2,2'-Methylene-bis-(6-(2H-benzotriazol-2-yl)-4-(1,1,3,3-tetramethylbutyl)-phenol)	Sigma-Aldrich	○	NC	Non-irritant	NT
44	118-82-1	4,4'-Methylene bis-(2,6-di-tert-butylphenol)	Sigma-Aldrich	○	NC	Non-irritant	NT
45	68610-92-4	Cellulose, 2-(2-hydroxy-3-(trimethylammonium)propoxy)ethyl ether chloride (91%)	Sigma-Aldrich	○	NC	Irritant	Reversible
46	14075-53-7	Potassium tetrafluoroborate	Sigma-Aldrich	○	NC	Non-irritant	NT
47	66170-10-3	Trisodium mono-(5-(1,2-dihydroxyethyl)-4-oxido-2-oxo-2,5-dihydro-furan-3-yl) phosphate	Sigma-Aldrich	○	NC	Irritant	

FUJIFILM Wako: FUJIFILM Wako Pure Chemical Corporation, TCI: Tokyo Chemical Industry Co., Ltd. AK Science: AK Scientific Inc., ○: Reference chemicals listed in TG492 performance standard (PS), NT: Not tested.

Table S2a. The outcome of the eye irritation evaluation using the iHCE-NY1 model test method (PIP: 1 day) for liquid and solid.

**All type of chemicals**	*In vivo* Category (UN GHS)		Number of chemicals
	Category 1, 2A and 2B	No Category	

***In vitro* prediction**	**Irritant (viability ≤70%)**	29	6	35
	Non-irritant (viability >70%)	3	9	12
Number of chemicals		32	15	47

Sensitivity: 90.6% (29/32), specificity: 60.0% (9/15), accuracy: 80.9% (38/47). PIP: post-incubation periods.

Table S2b. The outcome of the eye irritation evaluation using the iHCE-NY1 model test method (PIP: 1 day) for solid.

**Solid chemicals**		*In vivo* Category (UN GHS)		Number of chemicals
		Category 1, 2A and 2B	No Category	
***In vitro* prediction**	**Irritant (viability ≤70%)**	9	3	12
	Non-irritant (viability >70%)	0	4	4
Number of chemicals		9	7	16

Sensitivity: 100.0% (9/9), specificity: 57.1% (4/7), accuracy: 81.3% (13/16). PIP: post-incubation periods.

Table S3a. Performance metrics for assessment of the eye irritation evaluation using the iHCE-NY1 model test method for Liquid in accordance with OECD criteria and this assay for eye hazard identification.

**Liquid chemicals**		*In vivo* Category (UN GHS)		
		Category 1	Category 2A and 2B	No Category
***In vitro* prediction**	Strong irritant	**100% (4/4)**<br>**≥75%**	21.0% (4/19)<br>≤30%	37.5% (3/8)<br>≤5%
	Moderate or mild irritant	0% (0/4)<br>≤25%	**63.2% (12/19)**<br>**≥50%**	0% (0/8)<br>≤30%
	Non-irritant	0% (0/4)<br>≤5%	15.8% (3/19)<br>≤30%	**62.5% (5/8)**<br>**≥70%**

Balanced accuracy: 67.7 % (=21{4+12+5}/31). Lower number: OECD performance criteria [8].

Table S3b. The outcome of the eye irritation evaluation using the iHCE-NY1 model test method (PIP: by 14 days) for liquid and solid.

		In vivo Category (UN GHS)		
		Category 1	Category 2A and 2B	No Category
***In vitro* prediction**	Strong irritant	100% (8/8)	33.3% (8/24)	33.3% (5/15)
	Moderate or mild irritant	0% (0/8)	54.2% (13/24)	6.7% (1/15)
	No-irritant	0% (0/8)	12.5% (3/24)	60% (9/15)

Balanced accuracy: 63.8% (= {8+13+9}/47).

Table S3c. The outcome of the eye irritation evaluation using the iHCE-NY1 model test method (PIP: by 14 days) for solid.

**Solid chemicals**		*In vivo* Category (UN GHS)		
		Category 1	Category 2A and 2B	No Category
***In vitro* prediction**	Strong irritant	100% (4/4)	80.0% (4/5)	28.6% (2/7)
	Moderate or mild irritant	0% (0/4)	20.0% (1/5)	14.3% (1/7)
	No-irritant	0% (0/4)	0% (0/5)	57.1% (4/7)

Balanced accuracy: 56.3% (= {4+1+4}/16).
